# Distinctive Steady-State Heart Rate and Blood Pressure Responses to Passive Robotic Leg Exercise and Functional Electrical Stimulation during Head-Up Tilt

**DOI:** 10.3389/fphys.2016.00612

**Published:** 2016-12-09

**Authors:** Amirehsan Sarabadani Tafreshi, Robert Riener, Verena Klamroth-Marganska

**Affiliations:** ^1^Sensory-Motor Systems Lab, Department of Health Sciences and Technology, Institute of Robotics and Intelligent Systems, ETH ZurichZurich, Switzerland; ^2^Reharobotics Group, Spinal Cord Injury Center, Medical Faculty, Balgrist University Hospital, University of ZurichZurich, Switzerland

**Keywords:** rehabilitation robotics, robotic tilt table, orthostatic hypotension, functional electrical stimulation (FES), cardiovascular system, linear mixed models, parametric bootstrap

## Abstract

**Introduction:** Tilt tables enable early mobilization of patients by providing verticalization. But there is a high risk of orthostatic hypotension provoked by verticalization, especially after neurological diseases such as spinal cord injury. Robot-assisted tilt tables might be an alternative as they add passive robotic leg exercise (PE) that can be enhanced with functional electrical stimulation (FES) to the verticalization, thus reducing the risk of orthostatic hypotension. We hypothesized that the influence of PE on the cardiovascular system during verticalization (i.e., head-up tilt) depends on the verticalization angle, and FES strengthens the PE influence. To test our hypotheses, we investigated the PE effects on the cardiovascular parameters heart rate (HR), and systolic and diastolic blood pressures (sBP, dBP) at different angles of verticalization in a healthy population.

**Methods:** Ten healthy subjects on a robot-assisted tilt table underwent four different study protocols while HR, sBP, and dBP were measured: (1) head-up tilt to 60° and 71° without PE; (2) PE at 20°, 40°, and 60° of head-up tilt; (3) PE while constant FES intensity was applied to the leg muscles, at 20°, 40°, and 60° of head-up tilt; (4) PE with variation of the applied FES intensity at 0°, 20°, 40°, and 60° of head-up tilt. Linear mixed models were used to model changes in HR, sBP, and dBP responses.

**Results:** The models show that: (1) head-up tilt alone resulted in statistically significant increases in HR and dBP, but no change in sBP. (2) PE during head-up tilt resulted in statistically significant changes in HR, sBP, and dBP, but not at each angle and not always in the same direction (i.e., increase or decrease of cardiovascular parameters). Neither adding (3) FES at constant intensity to PE nor (4) variation of FES intensity during PE had any statistically significant effects on the cardiovascular parameters.

**Conclusion:** The effect of PE on the cardiovascular system during head-up tilt is strongly dependent on the verticalization angle. Therefore, we conclude that orthostatic hypotension cannot be prevented by PE alone, but that the preventive effect depends on the verticalization angle of the robot-assisted tilt table. FES (independent of intensity) is not an important contributing factor to the PE effect.

## 1. Introduction

**Figure 1:**
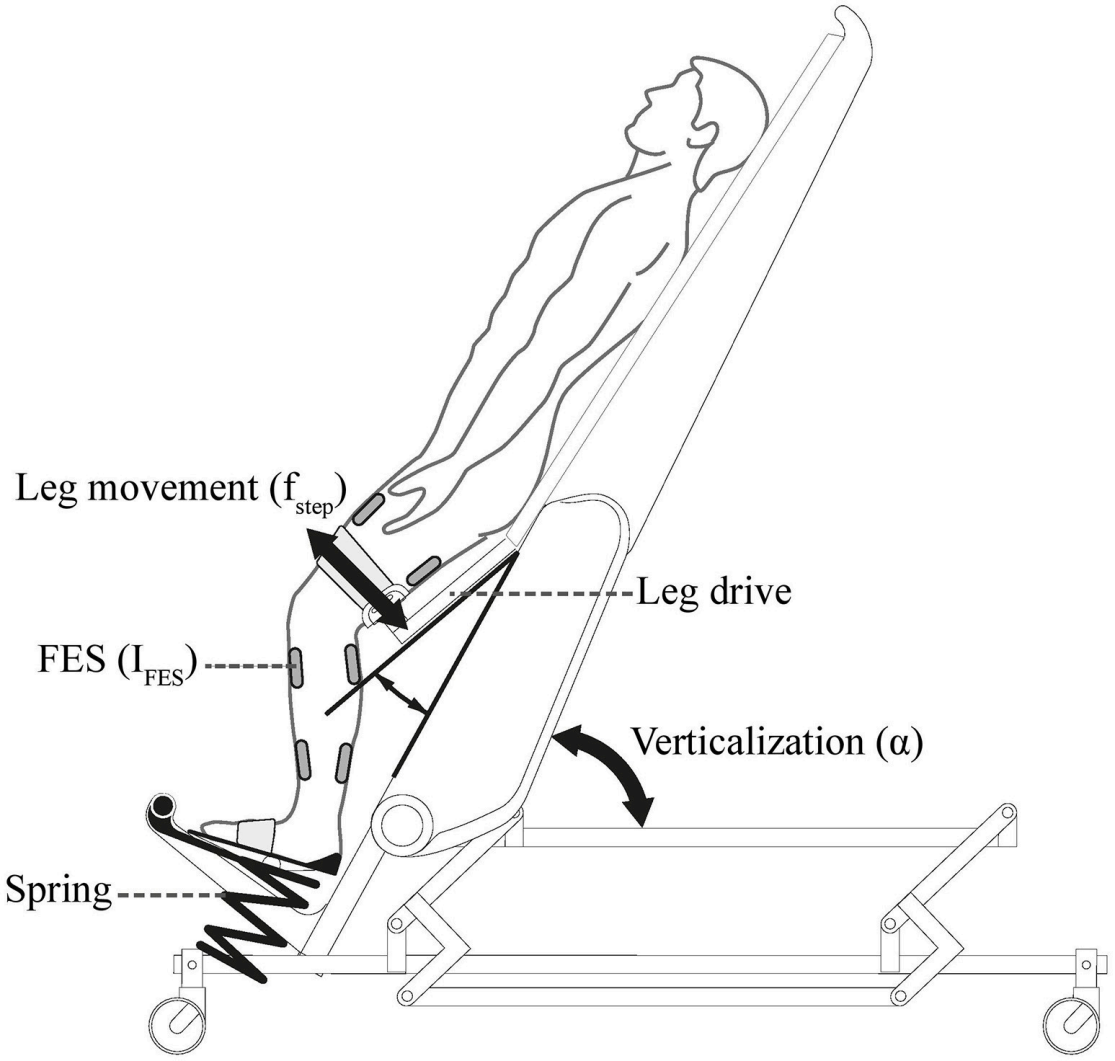
**Erigo tilt table: verticalization is provided by changing the inclination angle of the tilt table α**. Passive Robotic leg exercise is provided through a leg drive with an adjustable speed *f*_*step*_. The table is further enhanced with electrical stimulation module which enables providing electrical stimulation to the leg muscles with adjustable parameters (here current *I*_*FES*_) during robotic leg exercise. Picture is copyrighted by Hocoma AG, Switzerland, and is adapted with permission.

Diseases such as stroke or spinal cord injury often constrain patients to prolonged bed rest. This is often associated with negative secondary complications that might postpone or prevent recovery (Dittmer and Teasell, 1993; Brower, 2009). Mobilization averts such negative effects and might even promote the recovery (Morris, 2007; Burtin et al., 2009; Bourdin et al., 2010). However, it is challenging as many of the patients have orthostatic instability (Illman et al., 2000; Feldstein and Weder, 2012). A robot-assisted tilt table was proposed for safe mobilization of these patients (Colombo et al., 2005) and feasibility of its application in early rehabilitation of stroke and brain injury patients was proven (Kuznetsov et al., 2013; Frazzitta et al., 2015). Robot-assisted tilt table (Erigo, Hocoma AG, Switzerland) enables verticalization, passive robotic leg exercise (PE), and simultaneous provision of functional electrical stimulation (FES) to the leg muscles (Figure 1). It is believed that integration of the PE can enhance blood circulation and therefore, prevent orthostatic hypotension during head-up tilt (Czell et al., 2004; Colombo et al., 2005). This is because PE is consisted of passive robotic leg movements and cyclic leg loadings (provided by the springs beneath the subject's legs, see Figure 1), which result in improved muscle pump function and venous return, and thus, improved cardiovascular stability (Luther et al., 2008). The PE mechanism has also been used to develop biofeedback systems for early rehabilitation (Giggins et al., 2013), ranging from systems assuming complete passive inclusion of the subject in the biofeedback loop (Wieser et al., 2014; Sarabadani Tafreshi et al., 2015) to systems supposing active participation of the patient in the loop (Laubacher et al., 2015; Saengsuwan et al., 2015a,b).

It has been demonstrated that using robot-assisted tilt table in rehabilitation improves the orthostatic tolerance over time (Taveggia et al., 2015). Beside long-term efficacy, many studies have investigated the direct short-term effect of robot-assisted tilt table on orthostatic stability during rehabilitation and whether it helps to avoid orthostatic hypotension and consequent syncope during very early mobilization. In healthy subjects as well as patients, robot-assisted tilt table results in significantly less number of syncopes when compared to a normal tilt table (Czell et al., 2004; Luther et al., 2008; Kuznetsov et al., 2013). Thus, robot-assisted tilt table is currently being used in clinical practice with the aim to provide very early mobilization while avoiding orthostatic hypotension. The effect of the robot-assisted tilt table PE on blood circulation and cardiovascular parameters of healthy subjects (Chi et al., 2008) and patients (Yoshida et al., 2013) has been studied as well. However, almost all of these studies have been confined to explore the PE influence at one single tilt angle between 60° and 75°. Thus, is not clear yet whether the PE has the same influence at all tilt angles. Despite some preliminary investigations and assumptions (Wieser, 2011; Wieser et al., 2014), no systematic analysis has been performed. In this paper, we explore the effect of PE at different angles of head-up tilt. We hypothesized that the effect of PE on the cardiovascular system's response and therefore, its potential effect in preventing orthostatic hypotension, depends on the head-up tilt angle, at which the PE is performed. We also hypothesized that FES enhances the PE effect. To test our hypotheses, we analyzed the cardiovascular response of healthy subjects to different head-up tilt angles without PE, with PE, and including FES at different intensity levels.

Orthostatic hypotension is defined as a drop in systolic blood pressure (sBP) by more than 20 mmHg (in patients with supine hypertension 30 mmHg) or a drop in diastolic blood pressure (dBP) of more than 10 mmHg (Freeman et al., 2011). Since our goal was to evaluate the potential effect of PE on orthostatic hypotension at different tilt angles, we considered the changes in cardiovascular variables heart rate (HR), sBP, and dBP for the analysis.

The effect of head-up tilt alone on the cardiovascular system's response is well-described (e.g., Hainsworth and Al-Shamma, 1988; Lim et al., 2013). For comparative reasons and to provide more detailed information about the study subjects, we first analyzed the cardiovascular response of the subjects to head-up tilt maneuver. We then evaluated the effects of PE on the cardiovascular system's response. Finally, the effects of increasing the FES intensity during PE at different tilt angles was analyzed.

## 2. Materials and methods

### 2.1. Robot-assisted tilt table and measurement device

The robot-assisted tilt table (Erigo, Hocoma AG, Switzerland) (Figure 1) is offered for very early mobilization of bed-rest patients (Colombo et al., 2005). The tilt table inclination angle can be continuously changed between 0° and 75°. However, during our experiments we found the maximum effective tilt angle to be 71°. The motor-driven PE mechanism can move the subject's leg in a passive manner, and its speed can be adjusted between 0 and 80 steps/min. An FES module is also provided in the table which allows synchronized application of FES during PE to four main muscle groups in the leg (Mm. quadriceps femoris and tibialis anterior on the front side, and biceps femoris and gastrocnemius on the back side). FES parameters (amplitude, frequency, and pulse width) can be changed in real-time; FES current intensity (amplitude) can be adjusted for each channel separately, while adjustment of other parameters is non-channel specific.

We measured the raw blood pressure signal (100 Hz) non-invasively using a CNAP® monitor 500 (CNSystems Medizintechnik AG, Austria). The monitor uses a finger and arm cuff and requires an initial calibration time of about 2 min. The raw blood pressure signal was buffered online and its maxima and minima peaks were detected and averaged to calculate the real-time values of sBP and dBP, respectively. The HR signal was computed based on the heart period which was calculated using the time intervals between the dBP peaks. During all experiments, a sling was used to keep the subject's arm at the heart level.

### 2.2. Subjects and study protocols

We measured the HR, sBP, and dBP of 10 healthy subjects during four different study protocols. The study participants [10 males; mean age: 25.1 ± 2.6 years (standard deviation); mean weight: 81.0 ± 7.2 Kg; mean height: 181.2 ± 6.97 cm; mean body mass index (BMI): 24.8 ± 2.9] had provided written informed consent. The study was approved by the local ethical committee and was registered at ClinicalTrials.gov (registration identifier NCT02268266). The experiments were performed in a quiet room with normal temperature, and generally in 1 day. The subjects were asked to do not consume nicotine, alcohol, or caffeine 8 h before the experiments. Furthermore, they were asked to do not eat or drink (more than 1 dl) 1 h before each study protocol. Moreover, the participants were instructed to do not talk during the measurements (unless experiment termination would be desired).

**Figure 2:**
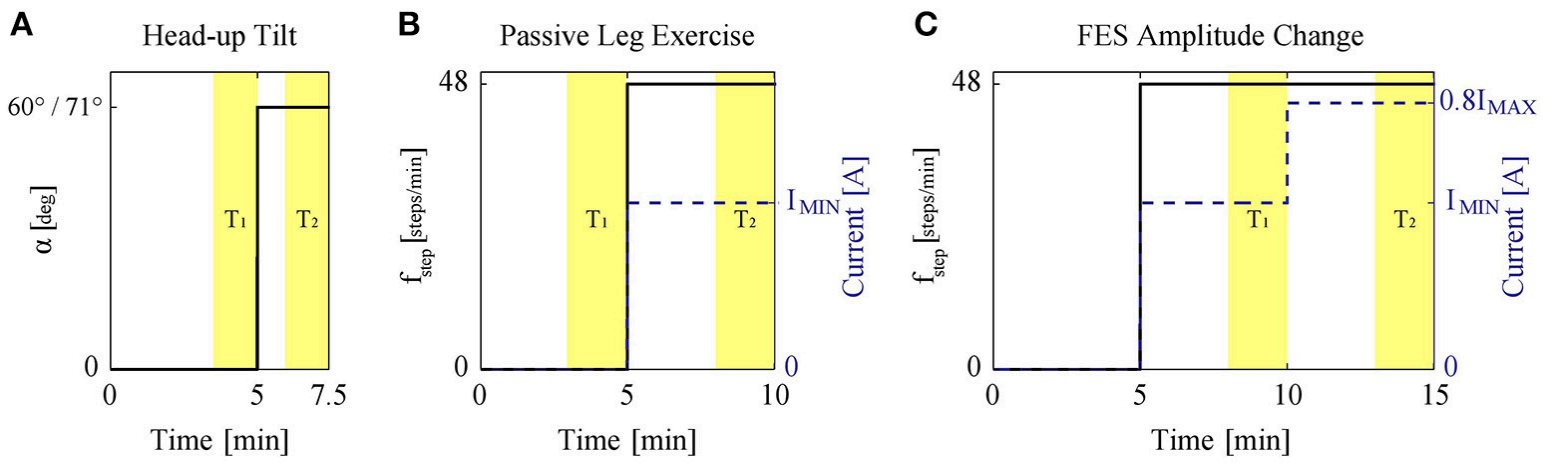
**(A)** Study protocol 1: head-up tilt to 60° and 71° (two experiments). **(B)** Study protocols 2 and 3: PE at 48 steps/min (solid) without or with application of the minimum FES amplitude (dashed). The protocols were conducted at α = {20°, 40°, 60°}. The figure shows the experiment performed at each specific tilt angle. **(C)** Study protocol 4: during PE at 48 steps/min (solid) with FES, the FES amplitude (dashed) was changed to a higher level, i.e., 0.8I_MAX_. The protocol was conducted at α = {0°, 20°, 40°, 60°}. The figure shows the experiment performed at each specific tilt angle. The highlighted areas *T*_1_ and *T*_2_ show the data range used for the analysis.

The original study protocols were longer. Here, we only present data related to the current study: (a) Study protocol 1: the measurement was started with a 5 min period of rest in supine position. Afterwards, the subject was tilted to α = 60° (Figure 2A). In a second variation, the same experiment was repeated but the subject was tilted to the maximum tilt angle (i.e., α = 71°) instead of α = 60° (Figure 2A). Thus, in this study protocol, two experiments per subject were performed.; (b) Study protocols 2 and 3: the subject was tilted to a specific tilt angle α = {20°, 40°, 60°} in a random order. Then, the measurement was started with a 5 min rest period in tilted position. Afterwards, PE at 48 steps/min for 5 min without FES (protocol 2) and with application of the minimum FES amplitude *I*_MIN_ (protocols 3) was performed (Figure 2B). Therefore, in total six experiments were performed for protocols 2 and 3.; (c) Study protocol 4: the subject was tilted to a specific tilt angle α = {0°, 20°, 40°, 60°} in a random order, where the measurement was started with a 5 min rest period in tilted position. Then, PE at 48 steps/min with minimum FES amplitude *I*_MIN_ was performed for 5 min (Figure 2C). The amplitude of FES was then increased to a higher level (i.e., 0.8*I*_MAX_) and together with PE continued for another 5 min. Accordingly, this study protocol included four experiments per subject.

To avoid any prior effects (e.g., due to measurement device calibration, etc.), all the experiments started with an initial 5 min rest period (Figure 2). Afterward, to study the different input types (i.e., head-up tilt, PE without FES, PE with FES, and FES amplitude), a step-like change in the input was applied. We considered the length of this step input such that steady-state response would have been reached. Most cardiovascular changes to head-up tilt take place during the first 30 s after tilting (Toska and Walløe, 2002). Thus, for protocol 1, a 2.5 min step input was considered enough to avoid the transients and reach the steady-state (Figure 2A). This 2.5 min period also included the time (about half a minute) to reach the tilted position.

We could not find any scientific study about the response time of PE and FES. However, based on our previous studies (Wieser et al., 2014) we expected that the time required to reach the steady-state response for changes in PE or FES, although longer than for head-up tilt, but to be <5 min (Wieser et al., 2014). Therefore, for protocols 2 and 3, after the initial 5 min rest, we considered performing PE for 5 min (Figure 2B). For protocol 2, PE was performed without FES, while for protocol 3, it was performed with minimum FES amplitude *I*_MIN_ (marked with a dashed line in Figure 2B). Similarly for protocol 4, where we wanted to study the effect of the change in FES amplitude, first we considered a 5 min rest period. Then, PE with minimum FES amplitude *I*_MIN_ for 5 min was performed to reach the steady-state response for this condition, and then after the change of FES amplitude, PE for another 5 min was carried out to ensure the steady-state response for the new condition with the new FES amplitude level (Figure 2C).

In study protocols 2–4, FES frequency was set to 40 Hz, FES pulse width to 300 μ*s*, and four muscle groups of the legs (i.e., Mm. quadriceps femoris, tibialis anterior, biceps femoris, and gastrocnemius) were stimulated. The stimulation was synchronized with the robotic leg movement, i.e., the quadriceps femoris and tibialis anterior muscles were stimulated during leg extension, while the biceps femoris and gastrocnemius muscles were stimulated during leg flexion. Before experiments, using a similar procedure as in Yoshida et al. (2013), we identified, for each subject and each muscle group, the minimum *I*_MIN_ and the maximum *I*_MAX_ current intensities. This procedure was performed once for each subject, in supine position, and took about 30 min. *I*_MIN_ was defined as the minimum FES current intensity *I*_FES_ producing a visible steady muscle contraction and *I*_MAX_ was defined as the maximum intensity tolerable by the subject. These values were in the range of 7–30 mA depending on the muscle and the subject. In the adjustment of stimulation intensity for each subject, although the muscle groups were treated differently according to their identified *I*_MIN_ and *I*_MAX_, one common intensity level was considered for all muscle groups. For example, the stimulation with *I*_MIN_ (e.g., Figure 2B) meant stimulation of all muscle groups with their already identified *I*_MIN_-values, while stimulation with 0.8*I*_MAX_ (e.g., Figure 2C) meant stimulation of all muscle groups with 80% of their already identified *I*_MAX_-values. Consideration of 80% threshold on *I*_MAX_ in study protocols was because of safety reasons.

To minimize the measurement time for the participants we restricted the tests for head-up tilt alone (protocol 1) to two independent experiments (at α = 60° and α = 71°), for PE without and with FES (protocols 2 and 3) to three independent experiments (for each at α = {20°, 40°, 60°}) and omitted testing the PE effect at α = 0° since we did not expect an effect in supine position due to missing afferent stimulation in the absence of feet loading (Wieser et al., 2014). However, for FES amplitude increase (protocol 4), as we expected that higher FES amplitude (independent of tilt angle) results in further muscle contraction and therefore, potential change in cardiovascular parameters, we also measured the effect at supine position α = 0°.

### 2.3. Data preparation

Initial postprocessing of the data was performed using Matlab 8.2 (Mathworks Inc., Natick, MA, United States). To study the effect of the external stimuli head-up tilt, PE without and with FES, and change in FES amplitude during PE on the steady-state values of the cardiovascular parameters—HR, sBP, and dBP– we calculated the steady-state values of the cardiovascular parameters before and during application of the external stimuli. To do this, we averaged the biosignals during two time intervals *T*_1_ and *T*_2_ (Figure 2), where we expected the steady-state response. Then we calculated the relative steady-state change for each cardiovascular parameter as:

(1) $$\begin{array}{r} \Delta Value=\mu_{2}-\mu_{1} \end{array}$$

where Δ*Value* corresponds to the relative change in the cardiovascular parameter [beats/min (bpm) or mmHg], and μ_1_ and μ_2_ correspond to the mean of the corresponding biosignal during *T*_1_ and *T*_2_, respectively. For study protocol 1, duration of *T*_1_ and *T*_2_ was 90 s, while for other study protocols it was 120 s (Figure 2). Since for protocol 1 (head-up tilt), we expected a shorter time to reach the steady-state response, this protocol was originally designed to be shorter (see above, Section 2.2). Thus, the considered *T*_1_ and *T*_2_ intervals for protocol 1 (90 s) were slightly shorter than other protocols, where similar as in (Wieser et al., 2014), 2 min periods were considered to calculate the steady-state values. The beginning of each interval was chosen such that the transient response in each case would be avoided and averaged biosignal value within the interval can be considered as a representative of the steady-state response.

### 2.4. Statistical analysis

For statistical analysis, we used the R-package (R Core Team, 2015) (version 3.2.0) to perform linear mixed model analyses (Bates et al., 2014). The statistical procedure was as follows:

1. **Model comparisons**: We started with an initial hypothesized model, used the maximum likelihood (ML) algorithm to fit models with various combinations of fixed and interaction terms to the data, and performed model comparison analyses using the Akaike information criterion (AIC) and Bayesian information criterion (BIC) scores to find which main effects and interactions are relevant in the initial model; those factors which were not relevant were excluded. Following this procedure, we found a model for how each cardiovascular parameter responded to the given stimuli.
2. ***P*-value calculation**: Calculation of *p*-values for linear mixed models due to ambiguity in the calculation of the denominator degrees of freedom for the test statistic is a controversial topic, and parametric bootstrap is proposed as a reasonable solution to construct and report *p*-values (Bates et al., 2014). To report the *p*-values for the final model we ran a parametric bootstrap (Bates et al., 2014) with 10,000 samples together with the ML algorithm. To do this, we excluded each relevant term once at a time and compared– using the likelihood ratio test (LRT) statistic and assuming it has a chi-square distribution—the reduced and full models. Each comparison yielded a chi-square value (observed LRT statistic). We then randomly simulated new data points under the fitted null hypothesis (i.e., under the fitted reduced model with one term less), refitted the reduced and full model on the new simulated data using the ML algorithm, and compared the two models to calculate a simulated LRT statistic value [parametric bootstrap (Bates et al., 2014)]. This procedure was repeated 10,000 times to generate 10,000 simulated LRT statistic values under the null hypothesis. Then the *p*-value for the fixed effect under examination was calculated as the fraction of the simulated LRT-values that are larger or equal to the observed LRT-value (Halekoh and Højsgaard, 2014). This procedure of calculating the *p*-values could not be applied in cases where, the final model (the outcome of Step 1) was the simplest model, i.e., a model only with an intercept term (the average of the data). In such cases, we bootstrapped coefficients for the intercept and used them to check whether the intercept is significantly different from zero. To this end, we ordered bootstrapped coefficients according to their values, calculated proportions of values larger (*p*^+^) and smaller (*p*^−^) than zero, and accordingly computed the *p*-value for the intercept as two times the minimum value of the proportions (i.e., 2 × *MIN*(*p*^+^, *p*^−^)). This was done according to the original *p*-value definition which states that for double tail events *p*-value is given by 2 × *MIN*{*Pr*(*T* ≤ *t*_*obs*_; *H*_0_), *Pr*(*T* ≥ *t*_*obs*_; *H*_0_)} where *Pr* stands for probability, *H*_0_ for null hypothesis, *T* is a continuous random variable, and *t*_*obs*_ is observed value (here, *t*_*obs*_ = 0) (Cox and Hinkley, 1979).
3. **Final model**: After calculating the *p*-values, we refitted the model using the Restricted Maximum Likelihood (REML) algorithm and we checked the normality of the residuals by inspecting Q–Q plot as well as performing a Shapiro-Wilk test (*p* < 0.05 significant violation) to ensure the validity of the model. The reported model parameter estimates and their standard errors (*SE*) are the outcomes of this stage.
4. **Confidence Intervals**: To calculate the confidence intervals (CIs) for each model parameter, we ran another parametric bootstrap with 10,000 samples on the final model (Bates et al., 2014).

The analyses for each cardiovascular parameter were done independently. We now present specific modeling considerations for each protocol.

#### 2.4.1. Protocol 1: head-up tilt alone

We performed linear mixed model analyses on the study protocol 1 data, to explore whether a general pattern can be observed in the steady-state response of the cardiovascular system to head-up tilt, and, whether these changes are different when subjects are tilted to slightly different tilt angles (i.e., α = 60° vs. α = 71°). To perform the linear mixed model analyses, we considered the tilt angle (α[*deg*]) as fixed effect into the model. However, to have an intercept (β_0_) that can be interpreted easier, we shifted the angle value with respect to α = 60°. Thus, the initially considered model for modeling HR, sBP, or dBP response to head-up tilt was:

(2) $$\begin{array}{r} \Delta Value=\beta_{0}\;+\;\beta_{1}\left(\alpha {}^{\circ}-60{}^{\circ}\right) \end{array}$$

where Δ*Value* corresponds to the relative change in the cardiovascular parameter value (bpm or mmHg), and β_*i*_s are model coefficients to be estimated.

As random effects, we considered a random intercept for each subject to account for potential correlations for repeated measures of the same subjects in two different versions of head-up tilt experiment [i.e., α = 60° and 71°] and furthermore, to account for variation in data due to random sampling of study subjects from the general population.

#### 2.4.2. Protocol 2+3: PE during head-up tilt

We examined whether a general pattern can be observed in the steady-state response of the cardiovascular system to PE and if yes, whether these changes are different when the PE is performed at different tilt angles (i.e., α = {20°, 40°, 60°}). Moreover, we tested whether FES of leg muscles during PE makes a difference in these changes. To this end, the data of the study protocols 2 and 3 were combined, and they were distinguished by considering a variable called “FES” representing FES status taking 0 (study protocol 2, i.e., without FES) or 1 (study protocol 3, i.e., with FES) values.

To perform the linear mixed model analyses, we considered the potential effect of tilt angle (α), FES, and interaction of angle effect and FES as fixed effects into the model. We shifted the angle term with respect to its value at α = 20°.

Graphical observation of data and results from previous studies (Wieser et al., 2014) suggested that the relationship between the change in cardiovascular variables in response to PE is non-linear with respect to the tilt angle α. Therefore, to account for a potential effect of angle non-linearity, various transformations of α were used in the initial model and compared. These, included the linear angle term [linear relationship, *f*(α) = α], the inverse of angle [*f*(α) = 1/α], the natural logarithm [*f*(α) = *ln*(α)], the angle square root [$f\left(\alpha \right)=\sqrt{\alpha }$], the angle squared [*f*(α) = α^2^], and *f*(α) = *sine*(α). The latter was motivated by the fact that the applied force on the feet of a subject (i.e., feet loading) during PE is proportional to the weight of subject times the sine of the tilt angle, and the PE effect on the cardiovascular parameters might be proportional to the feet loading (Wieser et al., 2014). Thus, the initial model we considered for HR, sBP, or dBP response to PE was:

(3) $$\begin{array}{r} \Delta Value=\beta_{0}+\beta_{1}\left[f\left(\alpha \right)-f\left(20{}^{\circ}\right)\right]+\beta_{2}FES\\ +\;\beta_{3}\left[f\left(\alpha \right)-f\left(20{}^{\circ}\right)\right]\times FES \end{array}$$

where Δ*Value* corresponds to the relative change in the cardiovascular parameter value (bpm or mmHg), β_*i*_s are model coefficients to be estimated, and *f*() is a transformation (i.e., $f\left(\alpha \right)=\left\{\alpha ,1/\alpha ,ln\left(\alpha \right),\sqrt{\alpha },\alpha^{2},sine\left(\alpha \right)\right\}$) performed on the tilt angle values α before fitting the model. For better numerical stability of the models the transformations *f*(α) = {1/α, α^2^} were applied to the tilt angle values in radians α[*rad*] while other transformations were applied to the angle values in degrees α[*deg*].

As random effects, we fitted individual intercepts for each subject to account for potential correlations due to repeated measures of the same subjects (at different tilt angles, and without and with FES) and to account for variation in data due to random sampling of study subjects from the general population.

Before entering the statistical procedure, we aimed at finding the best transformation *f*() of the angle term for each cardiovascular variable. To this end, the data were fitted by the ML algorithm considering various transformations of the angle term. For each transformation, all possible combinations of fixed and interaction terms present in the model were considered. This resulted in three models for each transformation. Then, the models with similar terms (but different transformations) were compared using the log-likelihood values to determine which angle transformation *f*() results in the best fit. Accordingly in the statistical procedure for each cardiovascular parameter, we considered the initial model with the corresponding best angle transformation.

#### 2.4.3. Protocol 4: FES amplitude change during PE

We used the study protocol 4 data, to investigate whether increasing FES amplitude during PE introduces significant systematic changes in the steady-state values of the cardiovascular parameters overall subjects and whether these changes are different at different tilt angles (i.e., α = {0°, 20°, 40°, 60°}). For the linear mixed model analyses, as fixed effect, we considered the potential effect of tilt angle (α) into the model. Similar to the protocol 2+3 data analysis (PE effect), we suspected that the relationship between the cardiovascular variables changes in response to the increase of FES amplitude would be non-linear with respect to the tilt angle α. To account for the potential non-linearity, beside the linear angle term [linear relationship, *f*(α) = α], we also considered the angle square root [$f\left(\alpha \right)=\sqrt{\alpha }$], the angle squared [*f*(α) = α^2^], and *f*(α) = *sine*(α). Accordingly, the initially considered model for HR, sBP, or dBP response to the increase of FES amplitude during PE was:

(4) $$\begin{array}{r} \Delta Value=\beta_{0}+\beta_{1}f\left(\alpha \right) \end{array}$$

where Δ*Value* corresponds to the relative change in the cardiovascular parameter value (bpm or mmHg), β_*i*_s are model coefficients to be estimated, and *f*() is a transformation (i.e., $f\left(\alpha \right)=\left\{\alpha ,\sqrt{\alpha },\alpha^{2},sine\left(\alpha \right)\right\}$) performed on the tilt angle values α before fitting the model. We applied all the transformations to the angle values in degrees α[*deg*], except *f*(α) = α^2^, which for the better numerical stability of the models, we applied it to the tilt angle values in radians α[*rad*]. Using a similar procedure as described above for the protocol 2+3 data analysis, the considered transformations of the angle term were compared using the log-likelihood values of corresponding models, to determine the best angle transformation *f*() for each cardiovascular parameter. For each cardiovascular variable, we then conducted the statistical procedure on the initial model with the best transformation found.

## 3. Results

### 3.1. Protocol 1: head-up tilt alone

**Figure 3:**
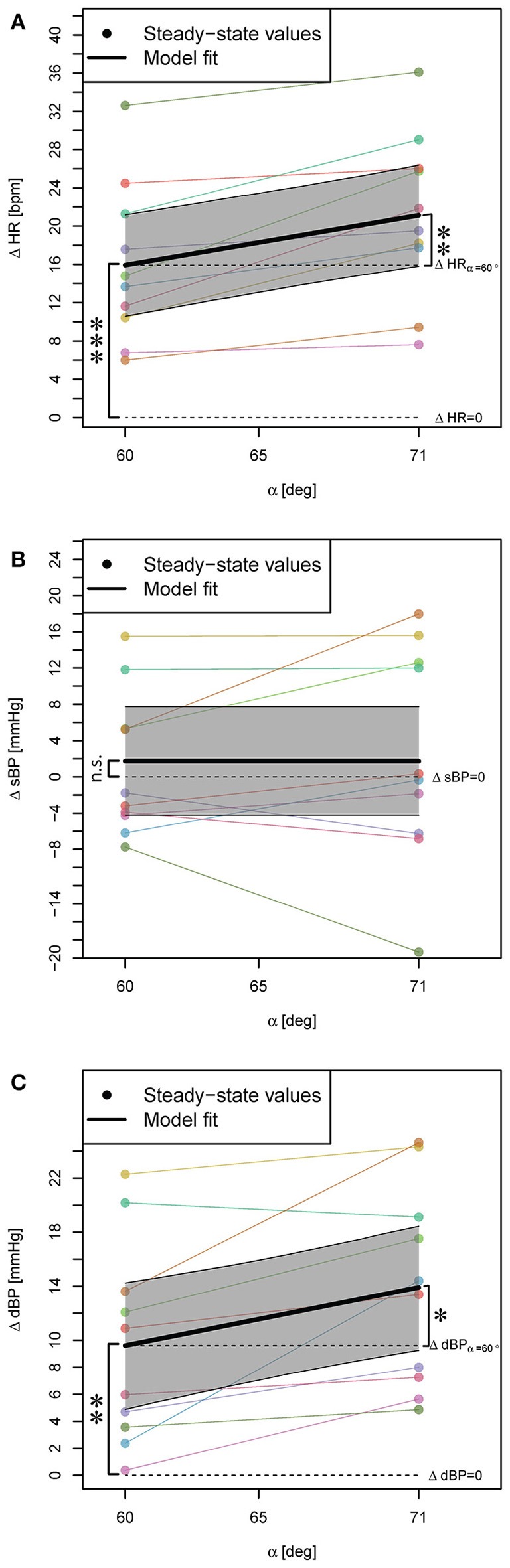
**Statistical models for ΔHR, ΔsBP, and ΔdBP responses (changes with respect to supine position) to head-up tilt alone (A–C)**. The steady-state values for each subject are connected with a line. The highlighted areas show 95% CI. The signs ^*^, ^**^, and ^***^ mark significant findings with *p* ≤ 0.05, 0.01, and 0.001, respectively. *n*.*s*. marks non-significant differences.

Head-up tilting to α = 60° resulted in a significant increase of HR and dBP (Table 1, β_0_, see also Figures 3A,C, respectively) but no change of sBP (Figure 3B). The result for HR and dBP was more pronounced by head-up tilting to α = 71° (Table 1, β_1_, see also Figures 3A,C), corresponding to 47 and 39% relative increases with respect to α = 60°, respectively. For sBP, there was no significant difference between α = 60° and α = 71° head-up tilting (irrelevant β_1_ coefficient in Equation (2) based on Step 1 of the statistical procedure).

**Table 1:** **Statistical models for ΔHR, ΔsBP, and ΔdBP responses to head-up tilt**.

**Cardiovascular variable**	**Parameter**	**Coeff**.	**Estimate**	***SE***	***t*-value**	**95% CI**	***P*-value**
ΔHR	*Intercept*	β_0_	15.92^***^	2.68	5.93	10.70, 21.12	0.0007
	(α° − 60°)	β_1_	0.47^**^	0.11	4.47	0.27, 0.68	0.0021
ΔsBP	*Intercept*	β_0_	1.74	3.02	0.58	–4.22, 7.74	0.5342
ΔdBP	*Intercept*	β_0_	9.60^**^	2.36	4.07	4.85, 14.23	0.0015
	(α° − 60°)	β_1_	0.39^*^	0.12	3.20	0.15, 0.63	0.0101

SE, standard error; CI, confidence interval. p-values are computed based on the parametric bootstrap and are unadjusted. The signs

*, **, and *** show significant findings with p ≤ 0.05, 0.01, and 0.001, respectively. Coeff. parameters represent the coefficients in Equation (2).

Of note: The subject with maximum HR increase (Figure 3A) also showed maximum sBP decrease (Figure 3B) [at 71° close to the 20 mmHg sBP decrease defined as sign of orthostatic hypotension (Freeman et al., 2011)]. This subject later reported that he had felt dizzy during the experiment.

### 3.2. Protocol 2 + 3: PE during head-up tilt

Comparison of the angle transformations showed that for the HR response, the transformation *f*(α) = 1/α, and for sBP and dBP responses the transformation *f*(α) = α^2^, result in the best fits.

**Figure 4:**
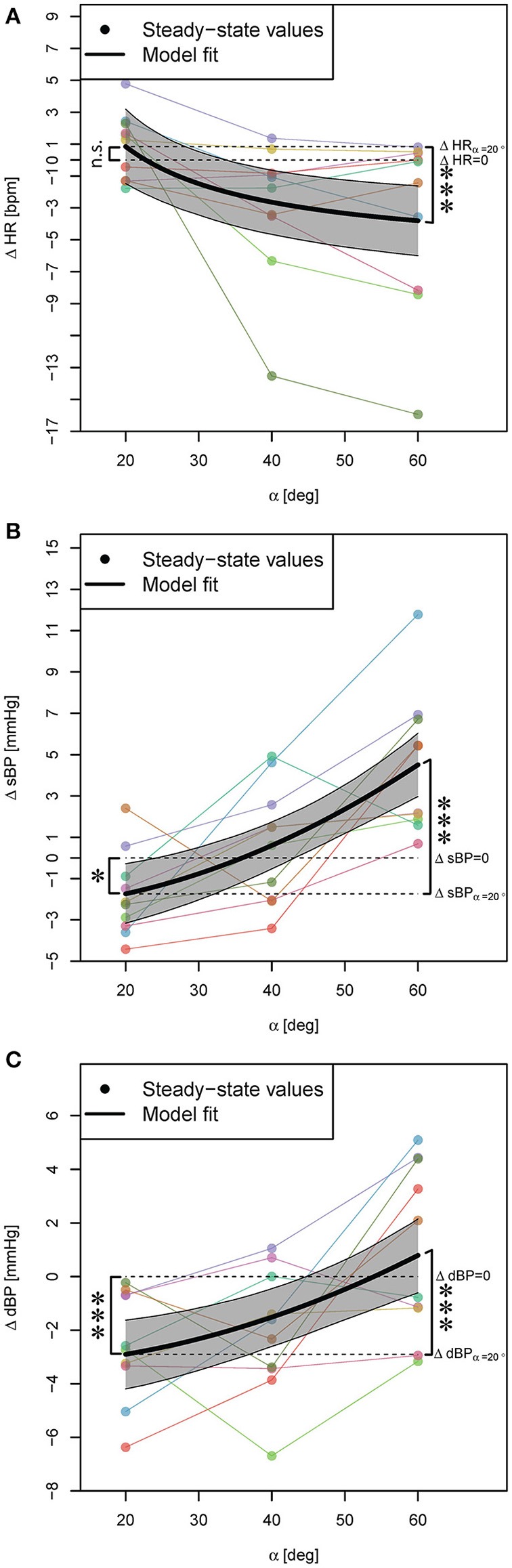
**Statistical models for ΔHR, ΔsBP, and ΔdBP responses to PE (independent of FES) during head-up tilt (A–C)**. Steady-state values correspond to the average response of two conditions, i.e., PE without and with FES application, and for each subject they are connected with lines. The highlighted areas show 95% CI. The signs ^*^ and ^***^ mark significant findings with *p* ≤ 0.05 and 0.001, respectively. *n*.*s*. marks non-significant differences.

FES did not have a significant contribution to the effect of PE on any of the cardiovascular parameters (irrelevant β_2_ and thus, β_3_ coefficients in Equation (3) based on Step 1 of the statistical procedure), and the outcome of the PE without and with FES was similar. Thus, the model (i.e., Equation 3) was reduced and included only β_0_ and β_1_ coefficients. Accordingly, protocols 2 and 3 were treated similarly and thus, in Figure 4, for better readability, the obtained steady-state values could be averaged and combined.

PE at α = 20° resulted in no significant change of HR (Table 2, β_0_, see also Figure 4A) but a significant decrease of sBP and dBP (Table 2, β_0_, see also Figures 4B,C, respectively). However, PE at higher head-up tilt angles significantly decreased the HR (Table 2, positive β_1_ for the inverse of the tilt angle, see also Figure 4A). This reduction of HR reached its maximum value at α = 60° (Table 2, ΔHR model, see also Figure 4A) which when compared to the HR increase in response to head-up tilt alone at the same angle (Table 1, ΔHR model, β_0_) amounts to about 24% reduction (i.e., about 4 bpm). In contrast to HR, sBP, and dBP significantly increased via PE by increasing the tilt angle (Table 2, β_1_, see also Figures 4B,C, respectively). Therefore, sBP and dBP responses to PE showed a change in the direction of the effect from negative to positive by increasing the tilt angle from α = 20° to α = 60° (Figures 4B,C, respectively). For sBP, this change in direction means that probably at some angle about α = 36° the PE has no effect on sBP (Table 2, ΔsBP model, see also Figure 4B). At α = 60°, PE results in a clear increase in sBP (Figure 4B). However, this increasing effect on sBP can be expected only at tilt angles above α = 45° (Figure 4B). For dBP, the change in the direction of the effect is rather a trend demonstrating a negative to zero effect of PE. PE at 20° and 40° introduces a reduction in dBP, and although it shows a trend toward having an increasing effect on dBP, it concludes with no significant effect at α = 60° (Figure 4C).

**Table 2:** **Statistical models for ΔHR, ΔsBP, and ΔdBP responses to PE without and with FES**.

**Cardiovascular variable**	**Parameter**	**Coeff**.	**Estimate**	***SE***	***t*-value**	**95% CI**	***P*-value**
ΔHR	*Intercept*	β_0_	0.85	1.21	0.70	−1.57, 3.24	0.4970
	$\left(\frac{1}{\alpha \left[rad\right]}-\frac{1}{20^{{}^{\circ}}\left[rad\right]}\right)$	β_1_	2.44^***^	0.60	4.03	1.23, 3.62	0.0002
ΔsBP	*Intercept*	β_0_	−1.74^*^	0.73	−2.38	−3.18, −0.31	0.0264
	(α[*rad*]^2^ − 20°[*rad*]^2^)	β_1_	6.41^***^	1.03	6.25	4.39, 8.46	0.0001
ΔdBP	*Intercept*	β_0_	−2.90^***^	0.67	−4.36	−4.21, −1.58	0.0010
	(α[*rad*]^2^ − 20°[*rad*]^2^)	β_1_	3.79^***^	0.85	4.47	2.12, 5.45	0.0001

SE, standard error; CI, confidence interval; rad, the corresponding angle value in radians. p-values are computed based on the parametric bootstrap and are unadjusted. The signs

*, and *** show significant findings with p ≤ 0.05, and 0.001, respectively. Coeff. parameters represent the coefficients in Equation (3).

Of note: The subject reported diziness during head-up tilt alone (see above) had the maximum HR reduction at α = 40° and α = 60° (more than 13 bpm).

### 3.3. Protocol 4: FES amplitude change during PE

The comparison of the angle transformations for this protocol showed that in general the transformation $f\left(\alpha \right)=\sqrt{\alpha }$ results in better fits. However, the angle term (i.e., β_1_ coefficient in Equation 4) was not relevant for any of the cardiovascular models (Step 1 of the statistical procedure). This resulted in models consisting of only intercepts, which later parametric bootstrap (Step 2 of the statistical procedure) showed that they are not significantly different from zero (Table 3). Thus, increasing FES amplitude during PE (independent of the tilt angle) did not result in any observable systematic changes in HR, sBP, and dBP.

**Table 3:** **Statistical models for ΔHR, ΔsBP, and ΔdBP responses to increase in FES amplitude during PE**.

**Cardiovascular variable**	**Parameter**	**Coeff**.	**Estimate**	***SE***	***t*-value**	**95% CI**	***P*-value**
ΔHR	*Intercept*	β_0_	−0.13	0.84	−0.15	−1.77, 1.51	0.8658
ΔsBP	*Intercept*	β_0_	−0.07	0.52	−0.13	−1.09, 0.95	0.8996
ΔdBP	*Intercept*	β_0_	−0.51	0.36	0.36	−1.22, 0.21	0.1552

SE, standard error; CI, confidence interval. p-values are computed based on the parametric bootstrap. Coeff. parameters represent the coefficients in Equation (4).

## 4. Discussion

### 4.1. Protocol 1: head-up tilt alone

The obtained results for head-up tilt alone are consistent with classical tilt-table studies that report the increase of HR and dBP by the head-up tilt with respect to supine position, and either increase (e.g., Chi et al., 2008; Wieser et al., 2014) or no change in sBP (e.g., Hainsworth and Al-Shamma, 1988). It is reported that the amount of this increase in HR and dBP has a linear relationship with the tilt angle between α = 20° and 60° of tilt (Hainsworth and Al-Shamma, 1988). However, our results similar to Wieser (2011) and Lim et al. (2013) show that the amount of increase after 60° becomes steeper (HR: 47%, dBP: 39% relative increase at 71°) and therefore, the relationship at higher tilt angles is non-linear.

Regarding the sBP response to head-up tilt, our results are in agreement with the studies which have found no significant change in sBP (e.g., Hainsworth and Al-Shamma, 1988). However, no significant change for sBP with respect to head-up tilt does not necessarily mean no change in sBP of the subjects. Some subjects experience an increase and some a decrease in sBP by head-up tilt (Figure 3B). These changes in two directions may cancel each other resulting in a non-significant intercept (change in sBP by α = 60° tilt). The change in sBP by varying the tilt angle from α = 60° to α = 71° was not significant as well. This observation might be because of rather small distance between these two tilt angles, which is not big enough to make a significant difference in sBP, or because the directions of the changes remain the same, and still cancel each other.

### 4.2. Protocol 2+3: PE during head-up tilt

Some previous studies have assumed that the effect of PE on the cardiovascular parameters is proportional to the sine of the tilt angle (Wieser et al., 2014). However, we found that for HR, the inverse transformation 1/α, and for sBP and dBP, the squared transformation α^2^ result in better fits. These results suggest that for HR, the outcome of PE at 40° and 60° are closer together while for sBP and dBP, the outcome of 20° and 40° are closer together. These imply that the effect of PE is not proportional to the feet loading, i.e., the amount of subject weight sensed by feet, but rather more complex neurophysiological circuits are involved, which play a more important role.

We observed that the cardiovascular system's response to PE differs at different tilt angles. At higher tilt angles than 20°, PE results in HR reduction, which suggests that PE at these tilt angles supports the heart in performing its objective. Furthermore, we observed that by varying the tilt angle from 20° to 60°, the direction of the effect of PE on sBP and dBP changes from reduction to increase of sBP and dBP values (Figures 4B,C). The PE increasing effect on sBP was only present at higher tilt angles than α = 45° (Figure 4B) suggesting that PE can potentially have a compensating orthostatic hypotension effect only at higher tilt angles. In particular, this compensating effect was observed in the subject who reported dizziness (see above, head-up tilt alone results). This subject at α = 60° head-up tilt demonstrated an sBP drop of about 8 mmHg, while PE at the same tilt angle resulted in an increase of sBP by about 7 mmHg. This implies that PE at 60° helps this subject to maintain orthostatic stability. In contrary to sBP, for dBP, we did not observe an increasing (i.e., compensating) effect at any tilt angles (Figure 4C).

Our findings are in contrast to the findings reported in Chi et al. (2008), where no significant effect of PE (independent of FES application) on HR, sBP, and dBP has been observed. Although, we did not observe a significant difference between PE without and with FES, we observed that PE in general has a significant effect on the cardiovascular parameters. Such a discrepancy between our findings and other studies might be the outcome of various differences between the studies (e.g., performing the PE at a different tilt angle or with a different intensity). Moreover, all previous studies (e.g., Chi et al., 2008; Yoshida et al., 2013) have explored the influence of the PE by comparing the effect of head-up tilting to one specific angle while participants are subjected to either PE or no PE. Thus, in these evaluations the head-up tilt and PE effect have always been considered together. In contrast to these studies, our study was designed such that the effect of PE and head-up tilt could be decoupled for independent considerations. This might have lead to a more precise evaluation of the PE effect.

### 4.3. Protocol 4: FES amplitude change during PE

Although, we did not observe any significant effect on the cardiovascular parameters by increasing FES amplitude during PE (see Table 3), it should be noted that the evaluation of the maximum tolerable intensity *I*_MAX_ in the subjects participating in this study was a subjective measure and a function of their pain tolerance threshold. Higher pain threshold, and therefore, higher FES intensities might not be an issue for some patients, for example, in individuals with complete paralysis. However, in comparison with healthy subjects, in such patients, with FES, less muscle force could be available due to inadequate muscle recruitment as a result of muscle atrophy in paralyzed limbs (Riener and Fuhr, 1998; Craven et al., 2013). Therefore, similar to healthy subjects, we do not expect that in these patients PE effect with higher FES intensities would be significantly different from PE effect with minimum FES *I*_MIN_. Since we also did not observe any significant difference between PE without FES and with minimum FES *I*_MIN_, thus, overall we do not expect that PE effect at higher FES intensities than *I*_MIN_ would be different than PE effect without FES. This is in accordance with Yoshida et al. (2013), where in patients, PE effect with FES at a higher intensity than motor threshold (i.e., *I*_MIN_ in our study), has not been found to be significantly different from PE effect alone.

### 4.4. Limitations

In the development of the statistical models for the cardiovascular response to each external stimulus, we treated each cardiovascular variable independently, although all biosignals were recorded during the same experiments. These variables are coupled through internal processes of the human body such as baroreflex circuit (Schwartz and Stewart, 2012), however, the degree of coupledness in response to each provided external stimulus is not clear, and therefore, it was not clear to what extent we should correct for the multiplicity. Thus, the reported *p*-values were not adjusted for multiplicity. Nevertheless, the effect of tilt angle on the cardiovascular parameters in response to head-up tilt alone as well as PE during head-up tilt, even with application of a conservative multiplicity correction method such as Bonferroni (multiplication of *p*-values corresponding to angle term by 3), is significant (*p* < 0.05) and re-confirms our findings regarding the dependency on the tilt angle in the corresponding analyses.

Finally, this study was performed on a small sample size of healthy subjects. How far the implications of our findings can be generalized to patients with cardiovascular disease or under complications that influence the cardiovascular system (e.g., prolonged bed rest; Adami et al., 2013) is unclear and should be investigated in future studies.

## 5. Conclusion and outlook

We proved that the effect of the PE of robot-assisted tilt table is strongly dependent on the tilt angle, and cannot be generalized to different tilt angles. Therefore, although PE might have a preventive effect on orthostatic hypotension, this preventive effect depends on the verticalization angle of the robot-assisted tilt table, at which amount of BP drop to head-up tilt alone becomes large enough to be a potential for orthostatic hypotension. Furthermore, in contrast to our hypothesis, FES independent of its intensity does not play an important role in the outcome of PE effect on the considered cardiovascular parameters in this study. In future, we will investigate the dependency of the PE effect on the tilt angle in a patient population.

## Author contributions

AS, RR, and VK conceived the study. AS designed and coordinated the study. AS collected and analyzed the data. AS, RR, and VK interpreted data. AS wrote the first draft of the manuscript. AS, RR, and VK revised the manuscript.

## Funding

This work was financially supported by the Commission for Technology and Innovation CTI, Switzerland, and the European Community's Seventh Framework Programme FP7/2007–2013 under grant agreement No. 312815—STAMAS.

### Conflict of interest statement

The authors declare that the research was conducted in the absence of any commercial or financial relationships that could be construed as a potential conflict of interest.

## Nomenclature

HR: Heart rate
sBP: Systolic blood pressure
dBP: Diastolic blood pressure
BP: Blood pressure
PE: Passive robotic leg exercise
FES: Functional electrical stimulation
ML: Maximum likelihood
AIC: Akaike information criterion
BIC: Bayesian information criterion
LRT: Likelihood ratio test
REML: Restricted Maximum Likelihood
SE: Standard error
CI: Confidence interval
